# Sex differences in climbing endurance emerge from combined physiological adaptation and neural strategies

**DOI:** 10.1007/s00421-026-06284-9

**Published:** 2026-06-06

**Authors:** Daniele Borzelli, Kamila Pstrag, Giuseppe Carollo, Beatriz Bachero Mena, Taian Martins Vieira

**Affiliations:** 1 Laboratory of Physiology, Department of Translational Medicine, Università del Piemonte Orientale, Novara, Italy; 2 Laboratory for Engineering of the Neuromuscular System, Department of Electronics and Telecommunications, Politecnico Di Torino, Corso Duca Degli Abruzzi 24, 10129 Turin, Italy; 3 Department of Human Movement and Sport Performance, University of Seville, Seville, Spain; 4 PoliToBIOMed Lab, Politecnico Di Torino, Turin, Italy

**Keywords:** Sport climbing, Muscle fatigue, Motor unit control, Neuromuscular strategies, Motor neuron control

## Abstract

**Background:**

Sport climbing imposes high endurance demands on finger flexor muscles, which sustain near-isometric loads for prolonged periods. Although women often demonstrate greater muscle endurance than men, the neuromuscular mechanisms underlying potential sex differences in climbing remain unclear.

**Purpose:**

Determine whether sex differences in climbing endurance are explained by physiological muscle adaptations, neural strategies of motor unit (MU) control, or both.

**Methods:**

High-density surface electromyograms (HD-sEMG) were recorded from the dominant forearm of 9 female and 13 male intermediate climbers during sustained body suspension on a campus board until failure, using two grip depths (20 and 30 mm) to manipulate task demand. EMGs were decomposed into MU discharge trains. Physiological adaptation was assessed via temporal changes in MU action potential amplitude and median frequency (MDF), while neural strategies were evaluated using traditional MU discharge metrics and a mode-based analysis of MU firing patterns.

**Results:**

Despite anthropometric differences, time to failure did not differ between sexes. MDF declined more slowly in women, indicating greater resistance to muscle fatigue. Traditional MU metrics showed greater discharge variability and intermittency in women. Mode analysis revealed three common temporal MU firing modes across sexes; however, men exhibited greater reliance on sustained early-phase MU activation, particularly under higher task demands.

**Conclusion:**

Sex differences in climbing endurance are not reflected in outcomes alone but arise from distinct neuromuscular mechanisms. Greater physiological fatigue resistance in women is complemented by sex-specific neural strategies of MU rate coding, underscoring the importance of integrating physiological and neural analyses when examining endurance performance in climbing.

## Introduction

Excelling in sport climbing requires high force production, resistance to fatigue, and fine motor control. Finger flexors are among the most stressed muscles, mainly due to the prolonged periods during which they sustain near-isometric contractions while supporting a substantial proportion of body weight (Limonta et al. 2016). Greater endurance in climbers, compared with untrained or less experienced individuals, has been documented in a growing number of studies (España-Romero et al. 2009; Ferguson & Brown 1997; Vigouroux & Quaine 2006). These studies point toward a specific physiological response of forearm muscles to climbing, rather than reliance on neural strategies to delay fatigue like the documented load sharing between motor units (MUs) or across muscles (Bawa et al. 2006; Kouzaki et al. 2004). Recently, for instance, we observed smaller changes in the median frequency (MDF) of high-density surface electromyograms (HD-EMGs) in experienced climbers during sustained body suspension until failure (Vieira et al. 2024). It is well established that the rate at which EMG descriptors change during an endurance task, the so-called myoelectric manifestation of fatigue, is commonly used as an index of muscle fatigability. Faster changes in EMG descriptors indicate greater susceptibility to fatigue and slower changes reflecting enhanced fatigue resistance (Gazzoni et al. 2017; Merletti et al. 1990). Using HD-EMGs, we were able to account for inter-individual differences in muscle size and for the uneven changes in muscle excitation, as the forearm region with greater changes in MDF varied markedly between individuals (Vieira et al. 2024).

Our previous findings prompt the question of whether well-known sex-differences in endurance (Berta et al. 2025; Hunter 2014; Maughan et al. 1986; Yoon et al. 2007) are explained by muscle adaptations, neural strategies to delay fatigue, or both, in climbers. Both physiological and neural mechanisms have been proposed to explain sex differences in muscle endurance. Evidence on the physiological mechanisms underlying these differences at the MU level is emerging. Women generally show a higher proportion of type I (slow‑twitch) muscle fibers, as well as greater capillarization and muscle oxygenation (Hunter 2014; Keller & Kennedy 2021) compared with men. These characteristics are associated with distinct contractile properties and may influence MU discharge behavior and fatigue resistance (Cidrais & Mendonça, 2026; James et al. 2025). Consistent with this, sex differences in MU behavior have been observed in multiple muscles: females tend to exhibit higher MU discharge rates at submaximal intensities and different firing strategies compared with males (Inglis and Gabriel, 2020). Also, lower‑threshold MUs in females seem to show greater neural drive and discharge rates during low‑intensity tasks (Olmos et al. 2023). Together, these findings suggest that peripheral muscle composition (fiber type) interacts with neural control mechanisms to shape sex‑dependent MU firing properties during sustained and submaximal contractions, supporting the idea that intrinsic physiological differences contribute to observed variations in motor unit activation between men and women.

However, evidence regarding neural mechanisms remains inconsistent and appears to be muscle-specific. For example, based on EMGs recordings from the biceps brachii muscle, Lecce et al (2024) observed a higher discharge rate in men for high-threshold MUs and in women for low-threshold MUs during submaximal isometric contractions. Similarly, in the vastus lateralis muscle, Guo et al. (2022) found higher discharge rates in females than in males at low contraction levels (< 25% of the maximal voluntary contraction; MVC). Inglis and Gabriel (2020) also reported a contraction level-dependence, with a steeper increase in discharge rate in men above 40% MVC. Corroborating this muscle-specific variability in MU discharge behavior, Peng et al (2018) and Parra et al (2020) reported greater MU discharge rates in women, respectively, in the vastus medialis and the first dorsal interosseous muscle. Considering the latter muscle, Nishikawa and colleagues (2024) reported higher discharge rates for MUs in women during both low (10%MVC) and high (60%MVC) contraction levels, along with greater recruitment thresholds.

While these studies consistently underscore sex differences in MUs’ discharge behavior, whether these differences extend to the modulation of MU discharge pattern (i.e., rate coding) remains unknown. The importance of analyzing MU rate coding, in addition to the traditional discharge statistics (e.g., mean discharge rate), lies in the existence of well-established neural strategies for delaying fatigue, including differential load sharing both between and within muscles (Bawa et al. 2006; Gazzoni et al. 2017; Kouzaki et al. 2004; Westgaard & De Luca 1999). Notably, most existing evidence has been obtained from isolated muscles under controlled conditions, and it remains unclear whether such differences translate to task-specific, whole-body performances such as climbing. In particular, it remains unknown whether the greater endurance typically observed in women arises primarily from peripheral physiological adaptations (i.e., reduced fatigability) or from sex-dependent neural strategies, such as differences in MU recruitment and rate coding. Addressing this gap requires simultaneously assessing physiological and neural determinants of MU behavior during a task that closely reflects the functional demands of climbing.

In this cross-sectional study, we assessed temporal changes in the action potential and discharge behaviour of MUs while male and female climbers performed sustained body suspensions until failure. We obtained MU data by decomposing HD-EMGs collected from the finger flexor muscles, with the goal of addressing the question: physiological adaptation, neural strategies, or both, which best explains the expectedly greater endurance of female climbers? Physiological adaptation, intended as increased resistance to fatigue, was assessed based on changes in EMG descriptors during the suspension task (Vieira et al. 2024; Vigouroux & Quaine 2006). Under this premise, action potentials of MUs in women would be expected to show smaller spectral and amplitude changes when compared with those in men. Neural strategies, in turn, were defined in terms of how rate coding is controlled in men and women. In such case, we expected to observe sex-differences in MU discharge pattern, with women exhibiting either out-of-phase variation in MUs discharge pattern (Bawa et al. 2006) or a predominant reliance on early recruitment of fatigue-resistance MUs (Guo et al. 2022). Our results show the neural strategies, more than physiological adaptation, to be a distinctive endurance mechanism between male and female climbers, with MUs rate coding showing opposite behaviour between groups.

## Methods

### Subjects

A total of 22 healthy climbers (9 females and 13 males) volunteered to participate in this study, with their anthropometric characteristics reported in Table 1. Participants were classified as intermediate climbers based on their reported performance level (from 6a to 6c + in the French sport climbing grading scale, ranging from 5 to 9a). All participants were informed of the experimental procedures and provided written informed consent prior to enrollment. None reported musculoskeletal injuries that could affect performance at the time of the experiments. Procedures conformed with the Declaration of Helsinki and were approved by the Ethics committee of Politecnico di Torino (98,992/2023).

**Table 1:** Anthropometric data (Mean ± SD values)

	Females (n = 9)	Males (n = 13)	P value
Age (yrs)	25.5 ± 4.5	29.8 ± 11.8	0.352
Height (m)	1.65 ± 0.07	1.77 ± 0.05	0.001
Body mass (kg)	56.5 ± 4.1	69.3 ± 5.0	< 0.001
Forearm_length_ (cm)	23.8 ± 1.1	26.3 ± 1.2	< 0.001
Wrist_circumf_ (cm)	15.1 ± 0.8	17.2 ± 0.6	< 0.001
Forearm_circumf 1/4_ (cm)	18.4 ± 1.4	21.8 ± 1.9	0.001
Forearm_circumf 1/2_ (cm)	23.2 ± 1.8	26.4 ± 1.8	0.002
Forearm_circumf 3/4_ (cm)	24.7 ± 1.4	28.2 ± 1.2	< 0.001

Detailed information on the protocol and data collection is provided in our previous study (Vieira et al. 2024). Here follows a brief description of the main experimental and data collection aspects.

### Experimental protocol

After 15-min warm-up, participants were requested to hold their body in suspension on a campus board using a half-crimp grip. The elbow joint was fully extended, and no trunk, head, or leg movements were allowed during the trial, which was continuously monitored by the experimenter. Failure was determined when contact was lost with the grip or when any gross movement was observed by the experimenter. From video recordings (cf next section), the experimenter determined the trial start as the instant when the participant achieved the required suspended position, while failure instant denoted the trial end. Three endurance trials were conducted, ordered according to the three-grip depths considered: 15 mm, 20 mm, and 30 mm. The order was fixed such that the shallowest grip depth (15 mm) was tested first, followed by 20 mm and then 30 mm, in order to minimize the influence of cumulative fatigue across conditions. At least 10-min of recovery was provided between consecutive trials. This recovery duration was chosen based on previous studies (Cornwall et al. 1994) and was considered sufficient to allow substantial recovery of force-generating capacity, particularly given the decreasing mechanical demands across trials (from 15 to 30 mm grip depth). Moreover, the fixed order ensured that the most demanding condition was always performed in a non-fatigued state; any residual fatigue would be expected to affect both sexes similarly.

Given that the duration of suspension trials in the 15 mm grip depth condition was shorter than 10 s in approximately half of the subjects tested, only the two thicker depths were analyzed. In addition to limiting the ability of the decomposition algorithm to reliably identify MU discharges (see below), such brief contraction durations do not allow for a meaningful assessment of fatigue-related neural strategies. By disregarding the 15 mm grip depth, we were also able to include subjects who had been discarded in our previous study (Vieira et al. 2024) due to their inability to suspend their body for more than 8 s.

### Electrodes’ positioning and EMG recordings

A flexible grid of 64 circular electrodes (8 rows, 1 mm diameter, 10 mm inter-electrode distance) was secured ventrally to the dominant forearm. Reference marks sought to standardize the positioning of the grid were drawn on the skin. Specifically, a reference line was drawn from the medial epicondyle to the midpoint between the radial and styloid processes. The grid was positioned with the reference line in the middle of the fourth and fifth columns, and with the most distal row of electrodes covering the forearm circumference drawn at 25% of the reference line (cf. Figure 1 in Vieira et al. 2024). A bi-adhesive pad filled with conductive paste (Ten20^®^ conductive paste, Weaver and Company) was used to secure the grid after cleaning the skin with abrasive paste (Nuprep^®^ Skin Prep Gel, Weaver and Company).

**Fig. 1:**
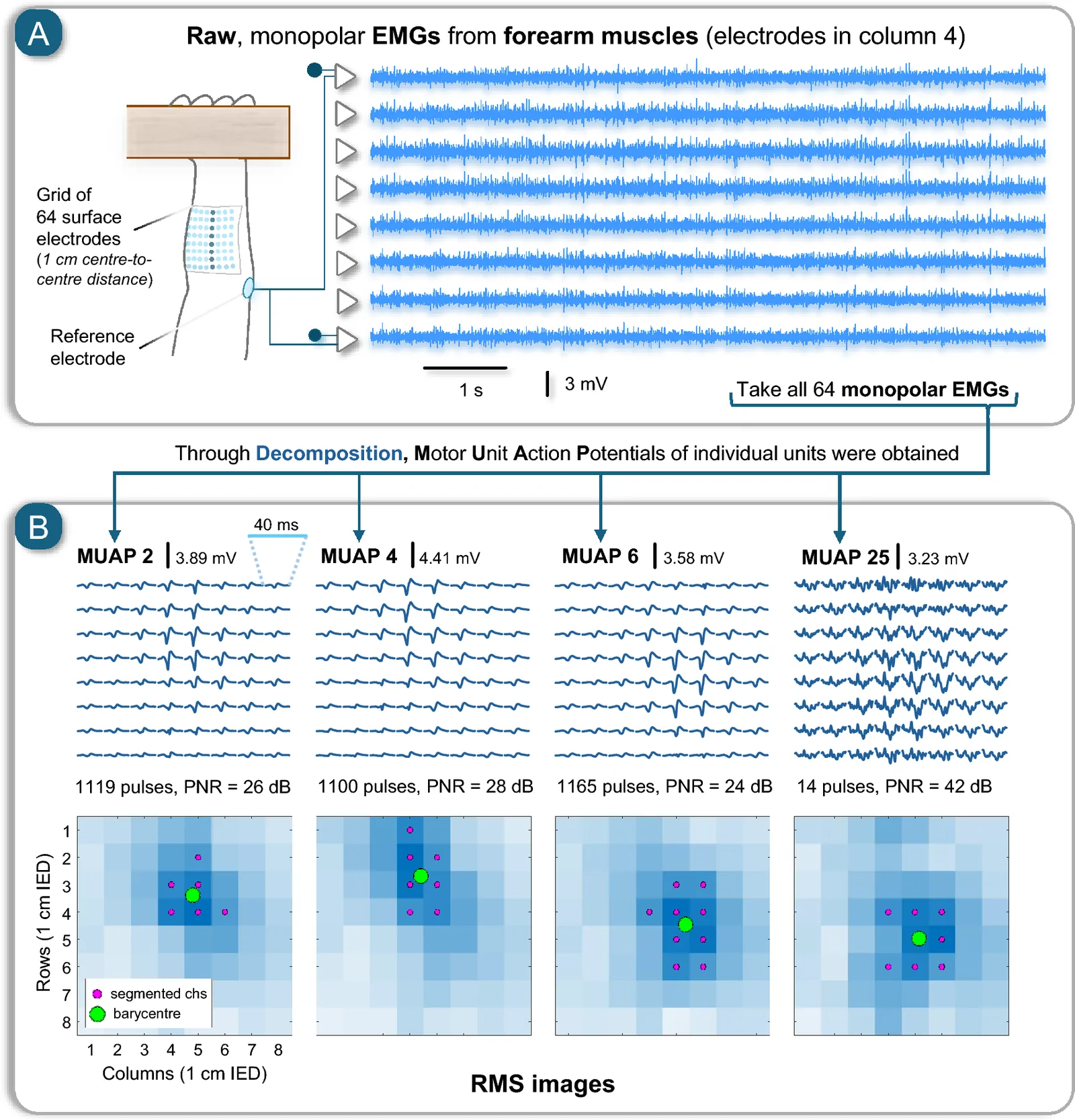
High-density surface EMG acquisition and motor unit decomposition. Panel A shows raw monopolar EMG signals recorded from forearm muscles using a 64-electrode grid (8 × 8, 1 cm inter-electrode distance). Signals from one electrode column are shown as an example. Taking four decomposed motor units as example, panel B shows their action potentials (MUAPs; top) and the corresponding root mean square (RMS) amplitude distribution (bottom). Motor units 2, 4, and 6 were retained for analysis, while motor unit 25 did not pass the quality assessment (cf EMG Decomposition). The number of discharges and pulse-to-noise ratio (PNR) are reported. Magenta dots indicate segmented channels, and the green circle denotes their centroid coordinates

Monopolar EMGs were recorded with a modular wireless system (Fig. 1A, Meacs, ReC Bioengineering and LISiN Politecnico di Torino, Italy). After 192 V/V amplification, EMGs were digitized at 2048 Samples/s with a 16-bit A/D converter. A 4 K full-body movie was recorded at 60 fps with a Samsung Galaxy S20 smartphone. EMGs and video recordings were synchronized offline using a common TTL trigger pulse sent to the auxiliary input of the EMG system and to a green LED visible within the camera field of view. EMGs were then trimmed from the first to the last video frame, defining the suspension trial.

### EMG decomposition

First, raw EMGs were visually inspected in single-differential configuration to identify low signal-to-noise ratio arising from contact issues, movement artefacts, or power line interference. Then, monopolar EMGs were band-pass filtered (20-400 Hz cutoff) and trimmed according to the trial start and end. With an automated and validated algorithm (Holobar and Zazula 2007), the trimmed monopolar EMGs were decomposed into the constituent trains of MU action potentials (Fig. 1).

Prior to analysis of MUs discharge and rate coding, the quality of decomposition results was assessed. MU action potentials waveforms were visually inspected, discarding those exhibiting dubious physiological validity (e.g. multiple innervation zones and spatial discontinuities, according to Power et al (2022), or pulse-to-noise ratio below 24 dB; cf. Discussion). Examples of retained and discarded MUs based on the quality of the waveform of their action potentials are provided in Fig. 1B. Subsequently, the discharge patterns of the remaining MUs were visually inspected. Discharges were screened to identify clear cases of missing discharges or false positives (Del Vecchio et al. 2020).

### Assessing physiological adaptations from MU action potentials

**Fig. 2:**
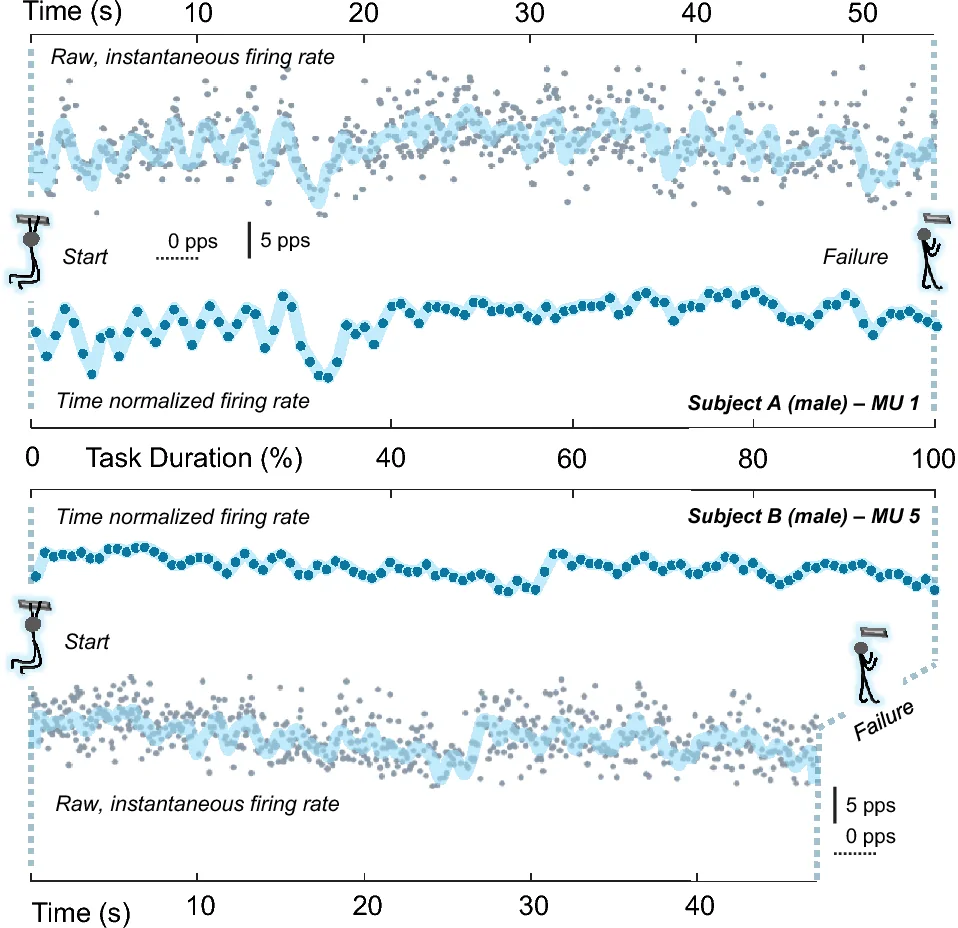
Motor unit action potentials across task duration. Motor unit action potentials recorded across the 64-channel electrode grid and averaged over the first, second, and third thirds of the suspension task. Waveforms are shown for each electrode position (1 cm inter-electrode distance). Darker traces indicate later task thirds. The dashed region highlights the segmented channels (cf. circles in Fig. 1B)

Amplitude and spectral changes of MU action potentials were considered to assess sex-differences in physiological adaptation of forearm muscles to climbing demands. According to the well-established concept of myoelectric manifestation of muscle fatigue, slower changes in EMG descriptors are associated with increased resistance to fatigue (Brody et al. 1991; De Luca 1984). Changes in EMG descriptors were analyzed only for the channels where each MU was mostly represented (Fig. 2). First, we used the discharges of each decomposed MU to segment (40 ms window), and then average EMGs (i.e., the spike triggered averaging procedure, Farina et al. 2003). This provides the surface representation of individual MU action potentials for each channel in the grid. For each averaged action potential, we computed the Root Mean Square (RMS) amplitude, yielding 64 RMS values arranged into an 8 *×* 8 fashion. Channels with RMS values exceeding 70% of the maximal RMS amplitude were considered to reflect the region most representative of the MU fiber activity (cf. segmented channels in Fig. 2): this 70% threshold was selected based on its optimal trade-off between false positives and false negatives (Vieira et al. 2010). These channels were therefore considered to assess the temporal changes in EMG amplitude and spectral descriptors. To assess temporal changes in MU action potentials across the task, each trial was divided into three equal-duration segments (i.e., first, second, and third thirds of the total suspension time, from task onset to failure). EMG descriptors and MU action potentials were then computed separately within each segment.

EMGs were processed for each MU only for the segmented channels (cf. circles in Fig. 1B). Short EMG windows (40 ms), centered on the firings of each decomposed MU, were considered for computing RMS and MDF values. For the MDF descriptor, the power spectrum was computed by squaring the magnitude of the EMG Fourier transform with a 1 s Hamming window, providing 1 Hz spectral resolution. One RMS value and one MDF value were therefore obtained for each MU discharge. Temporal changes in amplitude (RMS) and spectral (MDF) EMG descriptors were assessed from the slope of regression lines, computed in the least square sense for each of the considered channels and after normalizing RMS and MDF values by the first value (Vieira et al. 2024). Finally, slopes were averaged across channels, providing one slope value for each EMG descriptor and MU, RMS slope and MDF slope.

### Assessing neural strategies from the MU discharge pattern

Sex-differences in neural strategies were assessed by traditional metrics of discharge statistics and by a recent approach based on MU modes. Mean discharge rate and the coefficient of variation were computed for each MU after removing inter-spike intervals longer than 250 ms and shorter than 25 ms (Negro & Farina 2012). These metrics respectively indicate how strong the average neural input was and how much it fluctuated, not necessarily due to noise but to modulations in rate coding. We further assessed the degree of intermittency in MU activity by considering how often inter-spike intervals exceeded 250 ms in relation to the total number of inter-spike intervals (Vieira et al. 2012): greater intermittency was expected to denote more frequent episodes of MU decruitment and recruitment. Although these metrics are sensitive to average changes in rate coding, they are not informative about how these changes manifest during the fatiguing contraction. Cases of alternated modulation of discharge rate of different populations of MUs, for instance, cannot be appreciated from these traditional metrics.

**Fig. 3:**
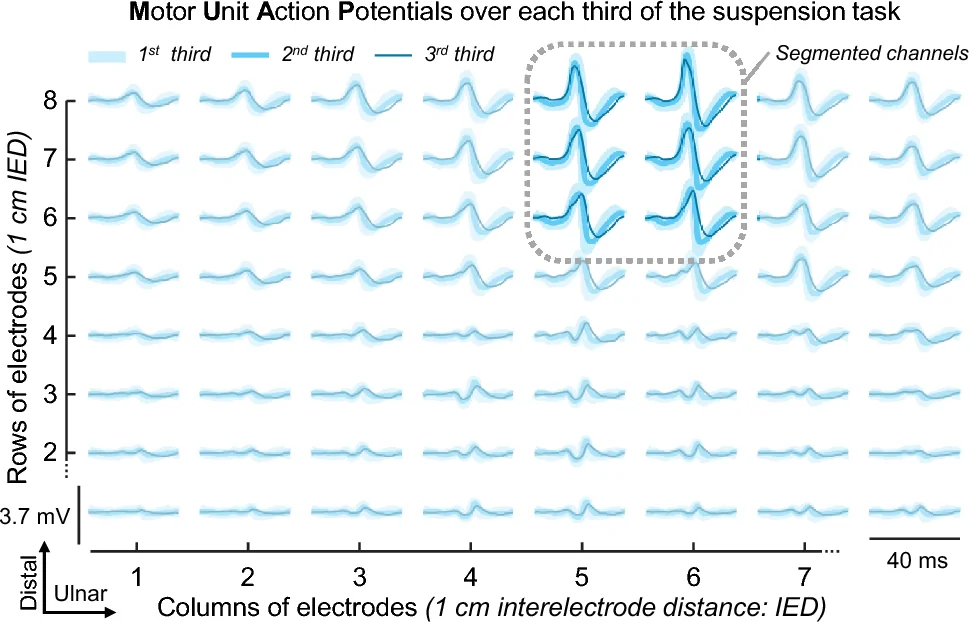
Motor unit firing rate during a sustained task. Raw instantaneous discharge rates (gray dots) and smoothed estimates (light blue) are shown from task start to failure for two representative motor units, from different subjects. Firing rates were time-normalized to task duration (dark blue), with the firing rate of all decomposed motor units spanning the whole task duration, from 0 to 100%. Dotted lines indicate task start and failure

To identify the main profiles governing the rate coding of MUs separately in male and female climbers, MU discharge profiles (modes) were extracted using the Principal Component Analysis, as recently proposed by Madarshahian and Latash (2022). Briefly, for each decomposed MU we created an *N*-element vector, with *N* being the number of samples from suspension start to failure (cf. Experimental Protocol). Each element in this vector assumed a value of either 1 or 0, according to whether there was a discharge or not. This train of sparse discharges was convoluted with a Hann window (1 s long), providing a smooth profile. Given *N* was different for each subject, we split each smoothed profile into 100 percentiles, each containing 0.1 *N* (rounded down) samples. Samples were averaged for each percentile, providing a time-normalized profile of the smoothed train of discharges, as schematically illustrated in Fig. 3. Each MU discharge profile was normalized by its maximum maximal discharge rate, so that the peak value equaled one. To prevent bias due to differences in the number of identified MUs across participants and conditions, the resulting profiles were then divided by the number of MUs identified for each participant and condition. The normalized MU discharge profiles were concatenated to form a matrix${\boldsymbol{X}}\in {\mathbb{R}}^{100\times {N}_{\mathrm{M}\mathrm{U}}}$, with 100 rows (normalized time) and ${N}_{\mathrm{M}\mathrm{U}}$ columns (total number of decomposed MUs).

The non-negative matrix factorization (Lee and Seung 1999) decomposed ${\boldsymbol{X}}$ as follows:

1 $${\boldsymbol{X}}={\boldsymbol{W}}{\boldsymbol{C}}$$

where ${\boldsymbol{W}}\in {\mathbb{R}}^{100\times {N}_{\mathrm{M}\mathrm{o}\mathrm{d}\mathrm{e}\mathrm{s}}}$ contained the common temporal modes, ${\boldsymbol{C}}\in {\mathbb{R}}^{{N}_{\mathrm{M}\mathrm{o}\mathrm{d}\mathrm{e}\mathrm{s}}\times {N}_{\mathrm{M}\mathrm{U}}}$ contained the modes’ coefficients, and ${N}_{\mathrm{M}\mathrm{o}\mathrm{d}\mathrm{e}\mathrm{s}}$ is the number of extracted modes. Each column of ${\boldsymbol{W}}$ thus represents how each mode evolves throughout the fatiguing task, while the *i*-th element of the *j*-th column of ${\boldsymbol{C}}$ indicates the contribution of the *i*-th mode to the discharge pattern of the *j*-th MU.

The quality of the reconstruction of ${\boldsymbol{X}}$ from ${\boldsymbol{W}}$ and ${\boldsymbol{C}}$ was quantified using the coefficient of determination (R^2^). For each number of modes (ranging from 1 to 25), 30 repetitions were performed with different random initializations, and the decomposition yielding the highest R^2^ was retained.

The optimal number of modes, ${N}_{\mathrm{M}\mathrm{o}\mathrm{d}\mathrm{e}\mathrm{s}}$, was determined as the smallest number that accurately captured the data variance, based on two criteria (Borzelli et al. 2013): (i) the R^2^ curve exhibited a change in slope, which was quantified by computing the mean squared error of a linear fit applied to the segment of the R^2^ curve between each number of modes, ranging from 1 to 24, and the maximum number tested (25), using a threshold of 1 × 10^–4^ and (ii) R^2^ exceeding 0.85.

### Statistics

Group differences in age, body mass, height, and forearm circumference and length were assessed with an independent sample t test after confirming the data Normal distribution. Main and interaction effects of group (females vs males) and grip depth (20 mm vs 30 mm) on time to failure, RMS and MDF mean slope, mean discharge rate, coefficient of variation, and Intermittency were assessed with a two-way analysis of variance test (Anova 2 × 2), after confirming the Normality of residuals with the Shapiro–Wilk test and using the Greenhouse–Geisser method to correct for unequal variances across levels of either factor when necessary according to the Levene’s test. Effect size was assessed through partial eta-squared (${\eta}_{p}^{2}$). The contribution of each of these MU metrics to failure time was assessed with multiple linear regression, considering the change in the sum of squared errors to identify the best subset of predictors (*stepwiselm* function in Matlab, with the sum of squared error criterion for including (*p* < 0.05) or removing (*p* > 0.10) terms from the model).

Differences in the use of each temporal mode between males and females, and across task efforts, were investigated using separate linear mixed models, one for each mode coefficient:

$${C}_{i}\sim gd*sex+gd+sex+\left(1|subj\right)$$

where ${C}_{i}$ represents the coefficients of the *i*-th mode. Fixed effects, grip depth (*gd*; *gd=0*: 30 mm depth; *gd=1*: 20 mm depth) and *sex* (*sex=0*: male; *sex=1*: female), and their interaction were tested. The participant index (*subj*) was included as a random effect to account for inter-subject variability. Statistical significance was assessed using Wald tests on the model coefficients (p < 0.05) (Fears et al. 1996). The sign of the estimated coefficient indicated the direction of the association.

## Results

### Anthropometric differences between groups and failure time

Groups were age-matched but differed significantly in all assessed anthropometric characteristics. On average, male climbers were roughly 13 kg heavier and 11 cm taller than female climbers, with forearm length and circumference being 3 cm greater (Table 1). Forearm volume was estimated by modelling the forearm as three truncated cones. Circumference was measured at three locations along the forearm (proximal, middle, and distal thirds) and at the wrist, and radii were derived from these measurements to compute the volume of each segment. Total forearm volume was obtained by summing the volumes of the three segments. Using this approach, female climbers exhibited a forearm volume about 32% smaller than that of male climbers.

**Fig. 4:**
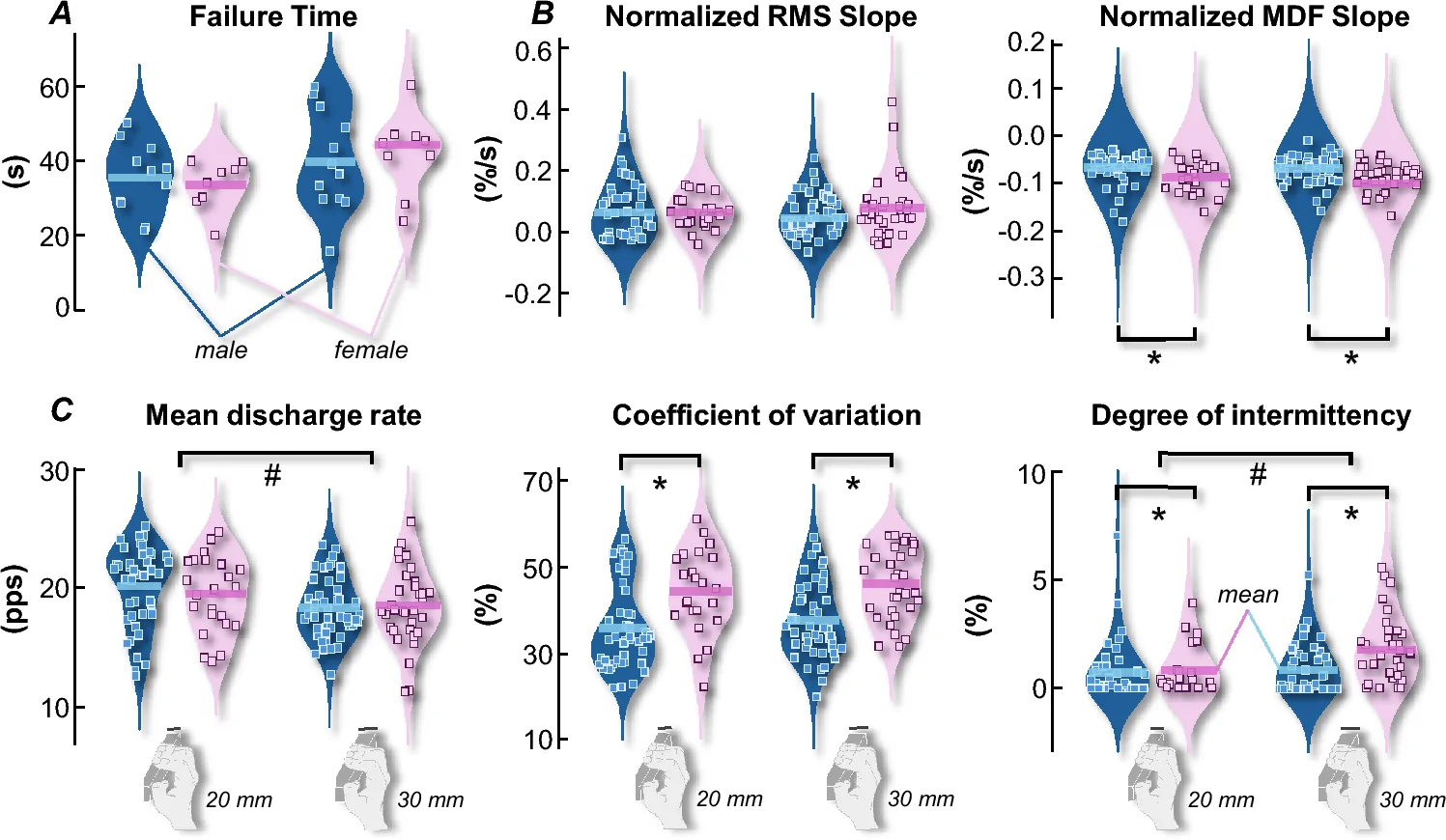
Sex- and condition-dependent differences in task performance and motor unit metrics. (**A**) Time to task failure for male and female participants. (**B**) Normalized slopes of EMG root mean square (RMS) amplitude and median frequency (MDF). (**C**) Motor unit discharge characteristics, including mean discharge rate, coefficient of variation, and degree of intermittency, are shown for the two task conditions (20 mm and 30 mm). Violin plots show distributions with individual data points overlaid. Asterisks and hash symbols indicate significant sex and condition differences, at p < 0.05

No differences in failure time were observed between groups, regardless of the grip depth considered (ANOVA main effect for group and interaction effect; *F* < 0.9, *p* > 0.41, ${\eta}_{p}^{2}$<0.06). There was a significant main effect of grip depth (*F* = 42.2, *p* < 0.001, ${\eta}_{p}^{2}$=0.74), with thinner grip depths resulting in shorter failure times (Fig. 4A).

### Myoelectric manifestation of MU fatigue

The number of MU decomposed differed between groups and grip depths. For the 20 mm grip depth, 19 and 47 MUs passed the quality assessment (cf *EMG decomposition* section in *Methods*) in female (n = 5) and male (n = 11) climbers, respectively. For the 30 mm grip, these values respectively increased to 32 (n = 8) and 51 (n = 10) MUs in females and males, respectively.

Variations in MU action potential waveform could be appreciated throughout the suspension task, mainly in terms of its duration. Figure 2 illustrates action potentials of a single MU, averaged across each third of the suspension task for a representative subject. As a first note, action potentials are represented in about 10% of the electrodes in the grid. Close inspection of waveforms for the segmented channels (shaded region in Fig. 2), reveals a visibly appreciable increase in the duration of action potentials when comparing the last and first thirds of the task.

EMG amplitude descriptor did not differ between groups or grip depths (Fig. 4B). No interaction (group *×* grip) or main effect was observed for RMS slope (F < 1.4, p > 0.23, ${\eta}_{p}^{2}$<0.01). Similarly, no interaction or main effect for grip depths on MDF slope was observed (F < 0.1, p > 0.76, ${\eta}_{p}^{2}$<0.01). However, regardless of the grip depth considered, MDF changed significantly slower in female climbers (main effect for group; F = 15.1, p < 0.001, ${\eta}_{p}^{2}$=0.1; Fig. 4B).

### Traditional MU discharge statistics

Group results are reported in Fig. 4C. Mean firing rate was greater for the shorter grip depth (*F* = 7.1, *p* = 0.008, ${\eta}_{p}^{2}$=0.05), but no group or interaction effect was observed (*F* < 0.7, *p* > 0.41, ${\eta}_{p}^{2}$<0.01). The coefficient of variation of inter-spike interval depended on sex, with female climbers showing average values nearly 10% greater than males for the two grip depths (*F* = 30.3, *p* < 0.001, ${\eta}_{p}^{2}$=0.17). The degree of intermittency was nearly dependent on both groups and grip depths (interaction effect: *F* = 3.67, *p* = 0.057), but a main effect was observed for each factor (*F* > 5.65, *p* < 0.019). Greater intermittency was observed for the thicker grip and for female climbers, with a low effect size though (${\eta}_{p}^{2}$<0.05).

### MU modes, groups, and grip depths

**Fig. 5:**
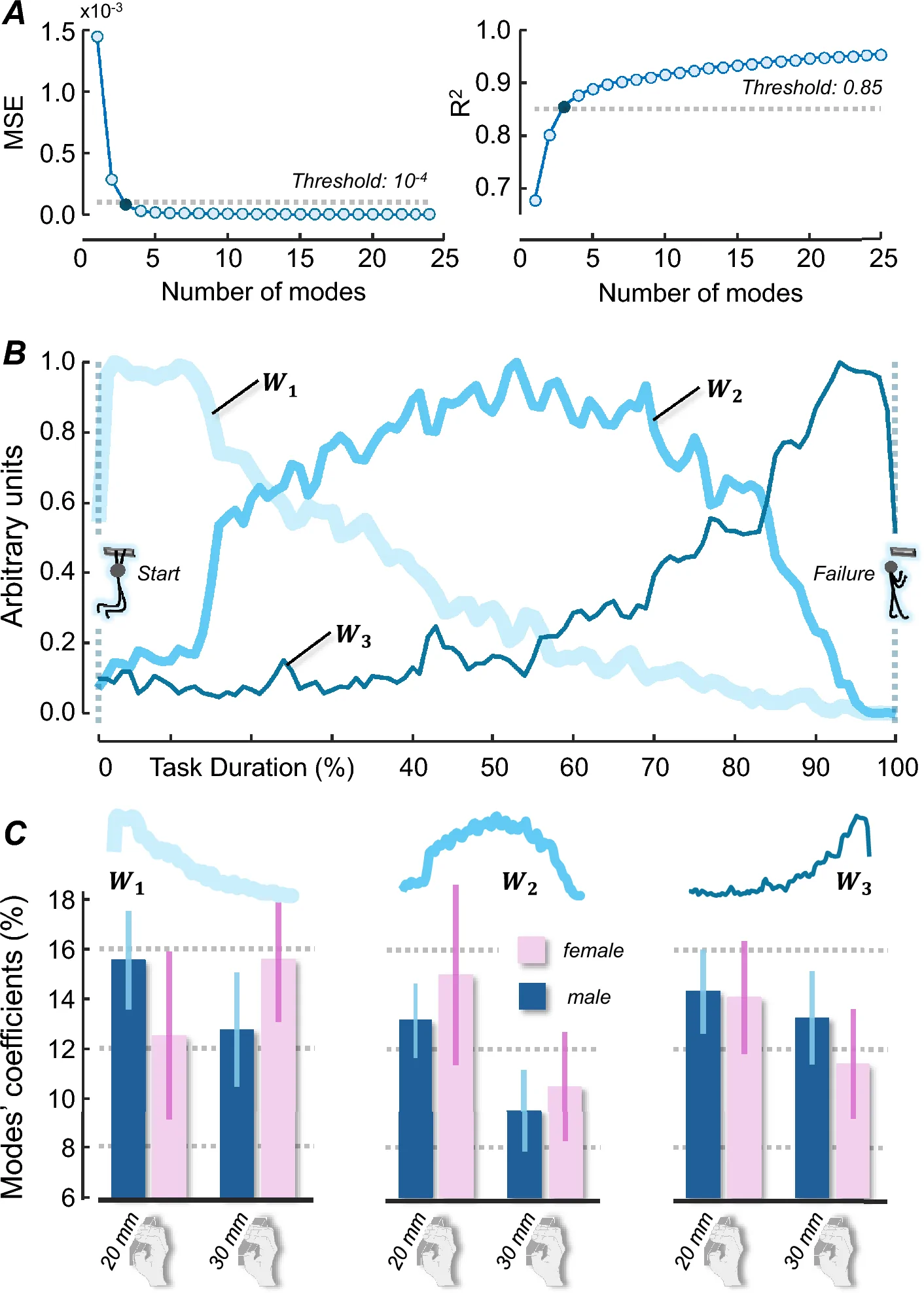
Mode selection and temporal evolution of task-related components. (**A**) Selection of the number of modes, based on the reconstruction of the mean square error (MSE) and explained variance (R^2^), with dashed lines indicating selection thresholds. (**B**) Temporal weighting coefficients of the selected modes (${{\boldsymbol{W}}}_{1}$–${{\boldsymbol{W}}}_{3}$) over normalized task duration, from task start to failure. (**C**) Relative contribution of each of the three modes shown in B, separately for each task condition (20 mm and 30 mm) and sex. Bars represent group means with standard deviation

A change in the slope of the R^2^ curve was identified for three modes, collectively explaining over 85% of the variability in MU firing patterns across participants and task conditions (Fig. 5A). Therefore, three temporal modes were retained for further analyses.

The three modes (${\boldsymbol{W}}$ in Eq. 1) exhibited distinct temporal behaviors during fatigue (Fig. 5B). The first temporal mode (${{\boldsymbol{W}}}_{1}$) showed a high contribution during the first ~ 12% of the suspension task, then progressively decreased toward failure. The second mode (${{\boldsymbol{W}}}_{2}$) rapidly increased its contribution to around 12% of the task and primarily contributed to the middle phase, peaking at approximately 50%, and then decreasing nearly the last decile. The third mode (${{\boldsymbol{W}}}_{3}$) exhibited an opposite pattern in relation to ${{\boldsymbol{W}}}_{1}$, progressively increasing its contribution and peaking near the end of the task (~ 92%).

Linear mixed model analyses, separately performed on the three temporal modes, revealed distinct effects (Fig. 5C). For ${{\boldsymbol{W}}}_{1}$, no main effect of grip depth (*p* = 0.22) or sex (*p* = 0.41) was observed, but a significant interaction between both indicated a lower mode weight in females for the 20-mm grip depth (*p* = 0.03). For ${{\boldsymbol{W}}}_{2}$, only a significant main effect of grip depth was observed, with the shorter grip resulting in greater mode weight (*p* < 0.01). Finally, ${{\boldsymbol{W}}}_{3}$ showed no significant effects of grip depth (*p* = 0.07), sex (*p* = 0.68), or their interaction (*p* = 0.77).

## Discussion

The present study aimed to clarify whether sex-related differences in endurance during sport climbing are primarily explained by physiological adaptations of the muscle, neural strategies of MU control, or a combination of both. By decomposing high-density surface EMGs recorded from finger flexor muscles during sustained isometric suspension to failure, we assessed sex-specific differences in myoelectric manifestations of fatigue and in MU discharge behavior. Despite marked anthropometric differences between male and female climbers, time to failure was similar across sexes. However, the underlying neuromuscular mechanisms differed: women exhibited slower spectral changes of MU action potentials and a more variable yet structured modulation of MU discharge rate. Together, these findings suggest that both physiological adaptations and neural strategies contribute to sex-related differences in endurance performance in climbing.

### Performance equivalence despite morphological differences

The similar failure times observed for men and women might, at first glance, suggest comparable endurance capacity. However, this interpretation requires caution. In the present protocol, neither the mechanical load applied to the finger flexors nor muscle size could be experimentally controlled. Male climbers were heavier and had substantially larger forearm dimensions, whereas female climbers had thinner forearms and, consequently, smaller muscle volume. Given that endurance performance is strongly influenced by the ratio between external load and active muscle mass, comparable failure times do not necessarily imply similar endurance abilities.

Indeed, the broader literature consistently reports greater endurance (fatigue resistance?) in women during sustained isometric contractions (Hunter 2014, 2018; Maughan et al. 1986; Yoon et al. 2007), particularly when contractions are performed at the same relative intensity. It is therefore plausible that, in the present study, female climbers sustained a relatively greater load per unit muscle mass than males. Matching male performance under such conditions would imply superior endurance-related adaptations, rather than true equivalence. This interpretation aligns with evidence indicating that women often exhibit enhanced fatigue resistance potentially due to a greater proportion of type I fibers and lower intramuscular pressures, favoring muscle perfusion during sustained contractions (Hunter 2014; Russ et al. 2005).

An additional aspect worth considering is the variability in failure time observed within each group. Although not the primary focus of the present study, such inter-individual variability may reflect differences in neuromuscular strategies. Previous work from our group and from others has shown that spatial and temporal features of EMG signals can be associated with endurance performance, with specific patterns of muscle activation correlating with time to task failure (Farina et al. 2008; Vieira et al. 2024). In the present study, we focused on group-level differences in physiological and neural mechanisms; however, exploring the relationship between individual EMG-derived parameters (e.g., MU discharge variability or mode weighting) and endurance time may provide further insight into subject-specific strategies. This aspect warrants dedicated investigation in future studies.

A methodological aspect to consider is the difference in anthropometric characteristics between male and female climbers. Although these differences reflect the natural variability between sexes and thus preserve the ecological validity of the task, they may also influence the interpretation of endurance performance and neuromuscular behavior. Matching participants for body size or strength across sexes would have allowed a more controlled comparison, but would also have reduced the representativeness of the sample. Similarly, although statistical approaches such as analysis of covariance could be used to adjust for anthropometric differences, the relatively small sample size and the imbalance between groups may limit the robustness of such corrections. Future studies specifically designed to disentangle the effects of sex and morphology, for example by matching participants or normalizing force output, would help to further clarify these aspects.

### Myoelectric manifestations of fatigue and physiological adaptation

Among the EMG descriptors considered, only MDF was sensitive to sex differences in myoelectric fatigue. Spectral descriptors are well known to be particularly responsive to fatigue-related changes, as they reflect slowing of muscle fiber conduction velocity associated with metabolite accumulation and altered membrane properties (Brody et al. 1991; De Luca 1984; Merletti et al. 1990). Consistent with this literature, MDF decreased more steeply in men than in women (Fig. 4B), regardless of grip depth.

Interpreted within the framework of myoelectric manifestations of fatigue, a slower MDF decline suggests greater fatigue resistance of the MUs recruited in female climbers. This finding is in agreement with our previous observations in climbers (Vieira et al. 2024) and supports the notion of a sex-specific physiological adaptation, potentially linked to differences in muscle fiber composition or metabolic profile (Carpentier et al. 2001; Hunter 2016). Importantly, no sex differences were observed in EMG amplitude changes, reinforcing the notion that spectral descriptors may be more sensitive indicators of endurance-related adaptations than amplitude-based metrics (Gazzoni et al. 2017).

### Traditional metrics point toward sex differences in motor unit discharge behavior

Traditional MU discharge metrics revealed clear sex-related differences in how neural input was modulated during the endurance task. Although the mean discharge rate did not differ between male and female climbers, women exhibited significantly higher coefficients of variation of inter-spike intervals and greater discharge intermittency (Fig. 4C). These findings indicate that, on average, female climbers relied on a more variable modulation of MU discharge rate than men.

Increased discharge variability has previously been associated with fatigue-delaying strategies based on rate coding rather than sustained activation of the same MU pool. Specifically, greater variability and intermittency have been interpreted as signatures of load-sharing mechanisms, whereby subsets of MUs modulate their discharge in counter-phase or undergo brief episodes of decruitment and recruitment to reduce metabolic stress (Bawa et al. 2006; Kouzaki et al. 2004; Westgaard & De Luca 1999). From this perspective, the higher coefficient of variation and intermittency observed in women suggest a greater reliance on such neural strategies, potentially contributing to their well-documented fatigue resistance during sustained isometric tasks (Hunter 2014). However, while these traditional metrics clearly indicate that MU discharge is more variable in women, they provide limited insight into how this variability is organized over time. Mean discharge rate, coefficient of variation, and intermittency are aggregate descriptors: they collapse MU behavior across the entire task and do not capture whether discharge variability follows a consistent temporal structure or reflects stochastic fluctuations. As such, these metrics alone cannot establish whether a characteristic, sex-specific neural strategy governs MU behavior during fatigue. This limitation motivated the analysis of MU discharge patterns using temporal modes. By decomposing the firing profiles into a small number of dominant components, the MU mode analysis allowed us to determine whether the observed variability reflects structured neural control strategies rather than unsystematic modulation.

A potential factor influencing MU discharge variability is the presence of small body oscillations during the suspension task. Although participants were instructed to avoid trunk, head, and leg movements, and trials were visually monitored to identify gross deviations from the required posture, subtle fluctuations in body position may still have occurred. Such fluctuations could lead to small variations in force output, potentially affecting MU discharge variability and intermittency. However, given the controlled experimental conditions and the absence of observable gross movements, it is unlikely that such effects systematically differed between sexes or fully accounted for the sex-related differences observed in MU behavior. Future studies combining EMG with direct kinematic or force measurements would help clarifying the contribution of mechanical fluctuations from neural control strategies.

### Motor unit modes reveal distinct endurance strategies, with different weights between sexes

The MU mode analysis provides direct insight into whether the sex-related differences observed in traditional discharge metrics reflect structured neural strategies. Three temporal modes were sufficient to explain most of the variance in MU firing behavior, and these modes were common to both sexes (Fig. 5A, B), indicating shared fundamental control mechanisms. However, their relative weighting differed between male and female climbers, revealing sex-specific modulation of neural drive.

The first mode (${{\boldsymbol{W}}}_{1}$) was dominant during the initial phase of the suspension task and progressively declined thereafter (Fig. 5B). This temporal profile is consistent with the behaviour of early-recruited, low-threshold MUs that initially contribute substantially to force production and subsequently reduce their discharge rate or become intermittently inactive as fatigue develops (Adam & De Luca 2005). Importantly, the contribution of ${{\boldsymbol{W}}}_{1}$ differed between sexes in the more demanding condition (20 mm grip depth), with female climbers exhibiting a lower weighting of this mode (Fig. 5C). This suggests that women relied less on sustained early-phase activation, consistent with a more conservative neural strategy aimed at preserving endurance. Such behavior aligns with the well-documented greater fatigue resistance in women during sustained isometric contractions (Hunter 2014). The second mode (${{\boldsymbol{W}}}_{2}$) peaked during the middle portion of the task and was primarily influenced by grip depth rather than sex. Its temporal profile likely reflects a redistribution of neural drive across the MU pool as fatigue progresses and task demands increase. Similar mid-task reorganization of MU activity has been linked to changes in common synaptic input and coordinated modulation of discharge rates during fatiguing contractions (Castronovo et al. 2015). The greater weighting of ${{\boldsymbol{W}}}_{2}$ for the smaller grip depth supports the notion that higher mechanical demands promote a shift toward more distributed control strategies, irrespective of sex. The third mode (${{\boldsymbol{W}}}_{3}$) emerged predominantly toward task failure, peaking near exhaustion. This late-phase component is consistent with compensatory neural mechanisms associated with increased central drive as contractile capacity declines (Enoka & Duchateau 2008). Although no sex differences were observed for ${{\boldsymbol{W}}}_{3}$, its presence underscores the progressive reorganization of MU control as fatigue develops.

Overall, the MU mode analysis demonstrates that the greater discharge variability observed in women is not random but organized according to specific temporal patterns. In particular, the sex-dependent modulation of ${{\boldsymbol{W}}}_{1}$ provides evidence of a distinct neural strategy in female climbers, complementing the physiological differences inferred from spectral EMG descriptors. Together, these findings support the conclusion that sex differences in climbing endurance may arise from the combined contribution of physiological adaptation and structured neural control strategies.

### Methodological considerations on motor unit decomposition

A methodological aspect deserving discussion concerns the PNR threshold adopted to identify valid MUs. The minimal PNR considered here (24 dB) is lower than the threshold commonly used in force-controlled isometric tasks (> 28–30 dB). This choice was motivated by the nature of the endurance task employed. During sustained suspension, force output was unconstrained and likely fluctuated due to alternation between muscles and MUs, as well as progressive slowing of action potentials with fatigue. Indeed, clear changes in MU action potential waveforms across task duration were observed (Fig. 2). Under such conditions, waveform variability can reduce PNR without necessarily compromising the physiological validity of the identified MU. Supporting this interpretation, Glaser and Holobar (2019) reported high sensitivity and low false-alarm rates for PNR values as low as 24 dB when decomposing EMGs during slow dynamic contractions. Given the additional visual inspection of MU waveforms and discharge patterns performed here, we are confident that the chosen PNR threshold did not introduce systematic bias and allowed retention of physiologically meaningful MU data.

## Conclusions

In summary, although male and female climbers exhibited similar failure times during sustained suspension, the underlying neuromuscular mechanisms differed. Slower MDF decline in women points toward greater physiological resistance to fatigue, while increased discharge variability and sex-specific weighting of MU temporal modes indicate distinct neural strategies of rate coding. These findings suggest that sex differences in climbing endurance cannot be attributed to a single mechanism. Instead, they emerge from the combined contribution of physiological adaptations and neural control, highlighting the importance of integrating MU-level analyses when investigating endurance performance.

## Data Availability

Datasets generated and/or analyzed during the current study are not publicly available but are available from the corresponding author on reasonable request.
